# Simultaneous Penile and Signet Ring Cell Bladder Carcinoma in Renal Transplant Recipient: A First Case

**DOI:** 10.1100/tsw.2009.108

**Published:** 2009-09-14

**Authors:** Francesca Manassero, Gianluca Giannarini, Davide Paperini, Andrea Mogorovich, Greta Alí, Ugo Boggi, Cesare Selli

**Affiliations:** ^1^ Department of Surgery, Section of Urology, University of Pisa, Italy; ^2^ Department of Surgery, Division of Anatomic Pathology, University of Pisa, Italy; ^3^ Division of Surgery in Uremic and Diabetic Patient, University of Pisa, Italy

**Keywords:** kidney transplantation, bladder cancer, penile cancer

## Abstract

The incidence and prevalence of cancer increase with time after transplantation. Therefore, a risk-adapted screening process is very important in order to identify low-grade malignancies early in their development. This provides the opportunity to initiate appropriate immunosuppressive regimens depending on the tumor type and stage of development. The first case presented is one of a 65-year-old patient with a double genitourinary carcinoma (penis and bladder). The patient received kidney transplantation 7 years prior to this event. After adequate surgical treatment (partial amputation of the penis for squamous cell carcinoma and complete transurethral resection of bladder adenocarcinoma), the patient was noted to be free of tumor recurrence and had functioning renal graft with a 2-year follow-up.

## INTRODUCTION

The increased survival either of transplant recipients or transplanted organs can increase long-term complications, in particular, malignancies. Different explanations have been advocated: immunosuppressive therapy can damage the DNA and interfere with usual mechanisms of restoration; viral infections with oncogenic power (Epstein Barr, hepatitis B, herpes, etc.) are quite common after transplantation or there is also loss of T suppression function, which acts against tumor induction and growth.

Regarding kidney transplantation, several studies have estimated a prevalence of cancer in recipients ranging from 4 to 18%[1]. The risk is three to five times greater than in the age and gender-matched general population, with lymphoma or skin cancer being the most predominant malignancies, followed by cancer of the urinary tract.

## CASE STUDY

A 65 year-old man and former smoker underwent kidney transplantation for chronic pyelonephritis from a deceased donor in July 1999. Induction therapy included basiliximab (20 mg pretransplant and 20 mg on day 4 post-transplant) and steroids. Maintenance immunosuppression consisted of a triple drug regimen based on steroids (quickly tapered to 5 mg/day), cyclosporine microemulsion (given at the initial dosage of 5mg/kg and subsequently adjusted as to maintain long-term blood trough levels between 125 and 175 ng/ml), and mycophenolate mofetil (the initial dose of 2 g/day was subsequently adjusted as to maintain white blood cell count above 3,000/mm$^3$, thus eventually averaging 1 g/day), without interruption during subsequent surgical procedures.

The urological screening consisted of an annual sonography of the native and transplanted kidneys, bladder, and prostate; PSA measurement; urinalysis and urinary cytology. Microhematuria prompted urological investigation and physical examination revealed a fungating lesion of the glans [Fig. 1].

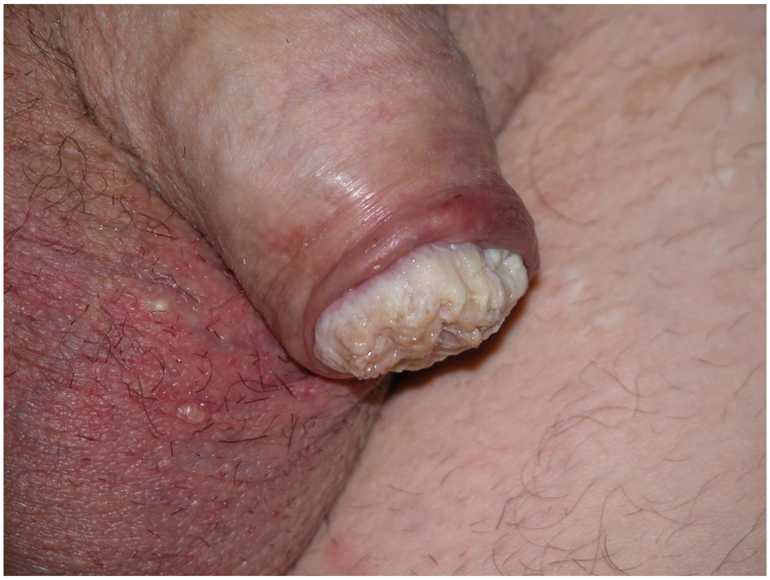

**FIGURE 1.** Macroscopic aspect. Squamous cell carcinoma of the glans penis, subsequently treated with partial penectomy.

In June 2006, the patient underwent partial amputation of the penis for squamous cell carcinoma (pT1G1Nx). *In situ* hybridization (ISH) for low- (6 and 11) and high-risk (16, 18, 31, 33, 35, 39, 45, 51, 52, 56, 58, and 66) human papillomavirus (HPV) types were performed on tissue sections, with negative results.

A careful inguinal physical examination and sonography was negative for enlarged lymph nodes. This evidence suggested that the patient had a low risk of developing nodal micrometastasis[2]; however, a meticulous surveillance program was decided by all the clinicians involved in the case.

During the same admission, the patient underwent cystoscopy to investigate an irregular bladder wall seen in the abdominal CT scan. The examination revealed a hyperemic suspicious area on the posterior bladder wall. A complete transurethral resection was performed and the pathological examination showed a signet ring adenocarcinoma of the bladder with marked proliferative activity (Fig. 2). Intracellular mucin was identified in signet ring cells with periodic acid-Schiff with diastase predigestion (PAS-D) histochemical stain. Random cold-cup biopsies of the remaining bladder mucosa were negative. An exploration of the gastrointestinal tract did not reveal any other tumor localizations, and endovesical chemotherapy with mytomicin C 20 mg/week for 6 weeks, then mytomicin C 20 mg/month for 11 months, was administered.

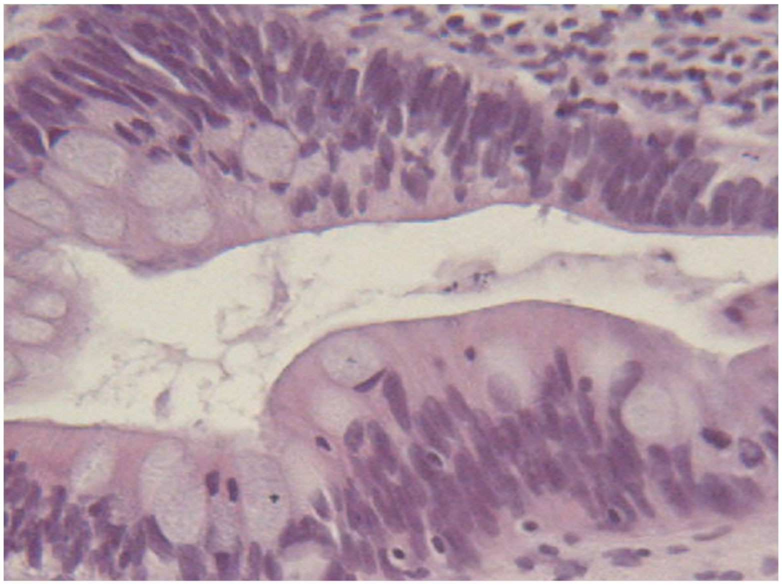

**FIGURE 2.** Histopathological aspect. Signet ring adenocarcinoma of the bladder. Hematoxylin and eosin ×200.

Two years later, both tumors were followed up according to EAU guidelines. The investigation highlighted a negative value.

The patient recently underwent laparoscopic cholecystectomy and removal of the infected right native kidney. During the same anesthesia procedure, a surgical inguinal staging biopsy of the sentinel lymph nodes did not reveal micrometastatic involvement, with two minimal vertical inguinal incisions. The graft function was not worsened by any operation (creatinine levels 1.2 mg/ml).

## DISCUSSION

The pattern of tumor growth in renal transplant recipients is very rapid. Tumor growth tends to be aggressive and to affect different organs. This results in a markedly decreased survival rate of the affected patients.

Most Western studies report that nonmelanotic cancer of the skin/lips and malignant lymphoma are the most common malignancies in transplant recipients, while a higher incidence of renal bladder cancers, hepatoma, gastric, and cervical cancers was observed in other studies.

Penile cancer accounts for up to 0.6% of all malignancies among men and some studies suggest an association with HPV infection. Despite a markedly increased incidence (3.5–17%) of epidermoid carcinoma in the transplant population, the first case of penile carcinoma in a renal transplant recipient was described only in 1979. Penile cancer represents only 6.3% of urological malignancies in the Oxford Transplant Centre registry[3,4].

When diagnosed in the early stages (T1-T2), penile cancer is highly curable and can be treated by conventional methods depending on the size, location, invasiveness, and stage of the tumor[4].

In a German study, a high incidence of transitional cell carcinoma and adenocarcinoma of the colon and liver was observed. In a British study, urogenital cancer was found to be the most common after skin cancer[4,5]. Several predisposing factors have been implicated in the development of bladder tumors in renal transplant recipients, including previous cyclophosphamide therapy and HPV infection (Type 11)[6,7].

Primary bladder adenocarcinoma is a rare tumor (0.5–2%), and the combination of primary bladder adenocarcinoma and renal transplantation is exceptional[8]. Primary signet ring cell carcinoma, described for the first time in 1955 by Saphir, is an uncommon histological subtype, accounting for approximately 0.24% of all bladder malignancies[9].

The majority of patients with primary signet ring cell carcinomas are diagnosed at an advanced stage and survival is poor. However, some patients with small tumors have been effectively treated by transurethral resection. Radiotherapy is ineffective in most cases and systemic chemotherapy is of no benefit[10].

To the best of our knowledge, this is the first case of primary signet ring cell carcinoma of the bladder in a kidney transplant recipient and also the first case of double urological cancer.

## CONCLUSIONS

Management of post-transplantation malignancies requires a multidisciplinary approach, aggressive treatment, and adjustment of the immunosuppressive regimens depending on the tumor type and its natural history. An important factor in the identification and appropriate treatment of early low-grade malignancies is a risk-adapted screening process.

Guidelines for screening and preventing solid-organ cancers in the general population should be strictly applied to renal transplant recipients. These patients are at greater risk of cancer, particularly of the genitourinary tract, and would benefit from aggressive intervention to reduce jeopardizing the graft survival.

## ACKNOWLEDGMENT

The authors thank Helen Badham for language revision.
